# Dielectric Elastomer Fiber Actuators with Aqueous Electrode

**DOI:** 10.3390/polym13244310

**Published:** 2021-12-09

**Authors:** Keita Shimizu, Toshiaki Nagai, Jun Shintake

**Affiliations:** Department of Mechanical and Intelligent Systems Engineering, Graduate School of Informatics and Engineering, University of Electro-Communications, 1-5-1 Chofu-gaoka, Chofu 182-8585, Tokyo, Japan; s2132048@edu.cc.uec.ac.jp (K.S.); n2032077@edu.cc.uec.ac.jp (T.N.)

**Keywords:** soft robotics, dielectric elastomer actuators, fibers, aqueous electrodes, underwater

## Abstract

Dielectric elastomer actuators (DEAs) are one of the promising actuation technologies for soft robotics. This study proposes a fiber-shaped DEA, namely dielectric elastomer fiber actuators (DEFAs). The actuator consisted of a silicone tube filled with the aqueous electrode (sodium chloride solution). Furthermore, it could generate linear and bending actuation in a water environment, which acts as the ground side electrode. Linear-type DEFA and bending-type DEFA were fabricated and characterized to prove the concept. A mixture of Ecoflex 00–30 (Smooth-On) and Sylgard 184 (Dow Corning) was employed in these actuators for the tube part, which was 75.0-mm long with outer and inner diameters of 6.0 mm and 5.0 mm, respectively. An analytical model was constructed to design and predict the behavior of the devices. In the experiments, the linear-type DEFA exhibited an actuation strain and force of 1.3% and 42.4 mN, respectively, at 10 kV (~20 V/µm) with a response time of 0.2 s. The bending-type DEFA exhibited an actuation angle of 8.1° at 10 kV (~20 V/µm). Subsequently, a jellyfish-type robot was developed and tested, which showed the swimming speed of 3.1 mm/s at 10 kV and the driving frequency of 4 Hz. The results obtained in this study show the successful implementation of the actuator concept and demonstrate its applicability for soft robotics.

## 1. Introduction

Soft robotics has recently attracted interest from several researchers [1,2,3]. Soft robots are made of compliant materials; they have excellent mechanical durability and adaptability to the external environment, as well as safety for humans. Soft robots are expected to find their application in exploration and rescue in natural environments, object handling in industry, and human assistance for rehabilitation purposes owing to these features.

Dielectric elastomer actuators (DEAs) are one of the promising actuation technologies in soft robotics [4,5,6,7]. DEA consists of an elastomer membrane sandwiched between two compliant electrodes. The opposing electric charges on the electrodes attract each other when subjected to a high voltage, squeezing the membrane in the thickness direction and expanding it in the planar directions. The structural simplicity of DEAs provides fast response speed (e.g., actuation at 600 Hz [8]), high electro-mechanical efficiency (theoretically up to 90% [9]), and large actuation strains (e.g., an area strain of more than 1000% [10]). The simple structure also allows tailoring the actuators in various configurations, e.g., by staking and rolling it to generate linear actuation and by attaching it to a flexible frame to output bending deformation [11,12,13]. Not only actuation but also the actuator can be used for self-sensing its deformations [14,15].

As a configuration of DEAs, fiber shape is promising given the studies based on other types of actuator technology. Fiber-type actuators provide multiple functionalities and high versatility in soft robotic systems [16], mimicking biological muscles [17] (active textiles [18]) and creating exoskeletons [19] and delicate grippers [20]. Therefore, fiber-shaped DEAs can be a versatile configuration, which enables diverse robotic systems.

**Figure 1:**
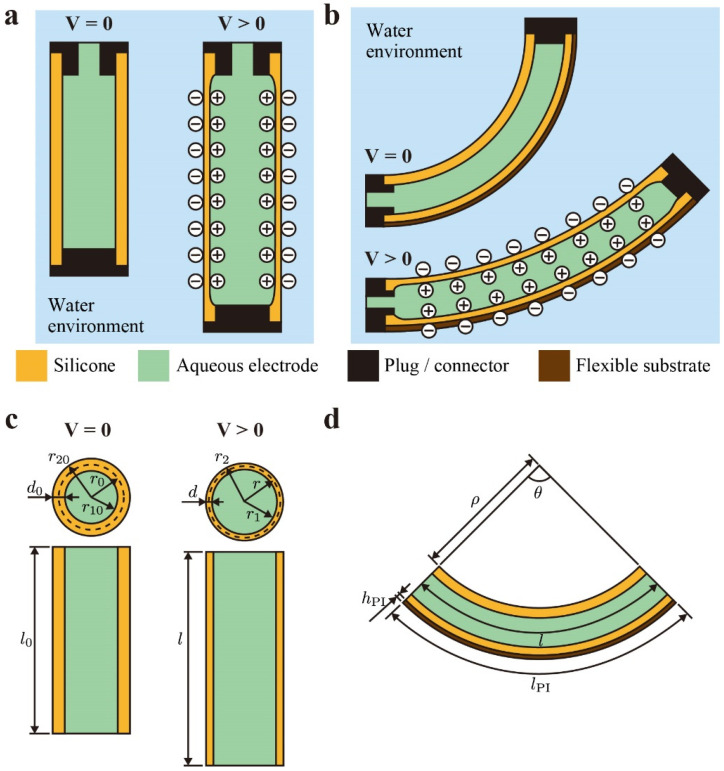
Working principle of (**a**) linear-type dielectric elastomer fiber actuators (DEFAs) and (**b**) bending-type DEFAs. (**c**) Schematics of linear-type DEFAs for modeling. (**d**) Schematics of bending-type DEFAs for modeling.

This study proposes dielectric elastomer fiber actuators (DEFAs). The actuator consists of a silicone tube filled with an aqueous electrode (in this study, sodium chloride solution), as shown in Figure 1a. The environment surrounding the device is water, which acts as the ground electrode. When a high voltage is applied to the aqueous electrode, the opposing charges are induced on the tube surface, resulting in a linear actuation. The surrounding water is often used as the ground electrode in other DEAs, especially in biomimetic underwater robots [21,22]. We used this actuation method with the aqueous electrode-filled tube because it can simplify the actuator structure, which would simplify the fabrication and modeling. There have been several studies on fiber-like DEAs in the literature [23,24,25,26]; however, none of these studies used aqueous electrodes and actuation in a water environment.

Our actuator DEFAs can also generate bending motion by applying a pre-stretch and attaching it subsequently to a flat flexible (but not extensible) frame (see Figure 1d), such as dielectric elastomer minimum energy structures [27,28]. Application of the voltage reduces the internal stress of the tube and bends the actuator toward the flat shape.

In the rest of this paper, we first construct an analytical model of DEFAs considering both the linear and bending actuation. After that, we fabricate and characterize actuator samples to clarify their actuation behaviors. Subsequently, we develop a jellyfish-type underwater robot and test it in a water environment to demonstrate the applicability of DEFAs for soft robotics. Finally, we discuss and conclude the results.

## 2. Actuator Model

We constructed an analytical model to predict and design the behavior of DEFAs. The linear actuation strain S or bending angle θ of the model was determined by calculating the local minimum of the potential energy based on the type of actuator, similar to other studies [27,29,30]. The potential energies include those of strain, electrostatic, bending, and tip load.

The silicone tube elongates in linear-type DEFAs when a voltage is applied (see Figure 1c). In this state, the deformation of the device is expressed as follows:

(1) $$l=l_{0}\lambda_{l},\;r=r_{0}\lambda_{r},$$

where l and r denote the length and radius of the tube and $l_{0}$ and $r_{0}$ denote their initial values, respectively. $\lambda_{l}$ and $\lambda_{r}$ represent the stretch ratio in the length direction and radius direction, respectively. r is the radius between the center and middle of the tube wall, as given in Equation (2).

(2) $$r=\frac{r_{2}+r_{1}}{2}=\frac{r_{20}+r_{10}}{2}\lambda_{r},$$

where $r_{2}$ and $r_{1}$ represent the outer and inner radius of the tube and $r_{20}$ and $r_{10}$ are their initial values, respectively. Similarly, the wall thickness d (i.e., the thickness of dielectric) is given as follows:

(3) $$d=r_{2}-r_{1}=\left(r_{20}-r_{10}\right)\lambda_{r}=d_{0}\lambda_{r},$$

where $d_{0}$ denotes the initial value of the wall thickness. The volume of the actuator was considered to be constant irrespective of any deformation due to the incompressibility of the silicone elastomer. Hence, the stretch ratios have the following relationship:

(4) $$\lambda_{l}\lambda_{r}^{2}=1,$$

Equation (4) is another expression of $\lambda_{r}$, i.e., $\lambda_{r}=1/\sqrt{\lambda_{l}}$. The strain energy of the actuator, $U_{\mathrm{strain}}$, can then be expressed as follows:

(5) $$U_{\mathrm{strain}}=\pi r_{20}^{2}l_{0}W\left(\lambda_{l}\right)=\pi r_{20}^{2}l_{0}\overset{3}{\underset{i=1}{\sum }}C_{i}{\left(\lambda_{l}^{2}+\frac{2}{\lambda_{l}}-3\right)}^{i},$$

where $W\left(\lambda_{l}\right)$ represents the strain energy density function. This study used the function from Yeoh hyper-elastic material model [31], involving $C_{i}$ as a material constant. We obtained $C_{i}$ by fitting Equation (6) to the tensile data (i.e., stress–strain curve) of the silicone elastomer (see Supplementary Materials for more details).

(6) $$\sigma_{l}=\lambda_{l}\frac{\partial W}{\partial \lambda_{l}}=2\left(\lambda_{l}^{2}-\frac{1}{\lambda_{l}}\right)\overset{3}{\underset{i=1}{\sum }}iC_{i}{\left(\lambda_{l}^{2}+\frac{2}{\lambda_{l}}-3\right)}^{i-1},$$

where $\sigma_{l}$ denotes the stress in the length direction (tensile direction). We assumed the entire structure to behave like a cylinder without a hollow made of silicone elastomer because the inside of the actuator was filled with the aqueous electrode, and both ends of the tube were sealed. A similar assumption was incorporated to model the bending stiffness of a silicone substrate encapsulating liquid-filled channels [32]. Therefore, the volume in Equation (5) considered the initial value of the outer radius, $r_{20}$.

The electrostatic energy, $U_{\mathrm{electric}}$, under an applied voltage V is defined as follows:

(7) $$U_{\mathrm{electric}}=-\frac{1}{2}CV^{2}=-\frac{\varepsilon_{0}\varepsilon_{r}\pi r_{0}l_{0}\lambda_{l}}{d_{0}}V^{2},$$

where C denotes the capacitance of the wall (dielectric), $\varepsilon_{0}$ is the permittivity of free space, and $\varepsilon_{r}$ represents the dielectric constant of the silicone elastomer. The sign of $U_{\mathrm{electric}}$ is negative because of considering the voltage-controlled case [19].

DEAs are often subjected to pre-stretch to orient their actuation behavior or adjust the initial deformation. One of the solutions for applying pre-stretch in our linear-type DEFAs was to put a tipping load. Hence, we considered the position energy of the load, $U_{\mathrm{load}}$.

(8) $$U_{\mathrm{load}}=-mgl_{0}\left(\lambda_{l}-1\right),$$

where m and g represent the mass of the load and gravitational acceleration, respectively. The sign of $U_{\mathrm{load}}$ is negative because the work was completed by the load. The total potential energy of the actuator, $U_{\mathrm{total}}$, was given as the sum of all energies expressed in Equations (5), (7), and (8).

(9) $$U_{\mathrm{total}}=U_{\mathrm{strain}}+U_{\mathrm{electric}}+U_{\mathrm{load}}.$$

The following equation is true for $U_{\mathrm{total}}$ under the equilibrium state:

(10) $$\frac{\partial U_{\mathrm{total}}}{\partial \lambda_{l}}=0.$$

Solving Equation (10) equals finding the local minimum of the total energy, which gives $\lambda_{l}$ as a function of V. Finally, the actuation strain S is calculated as follows: $S\left(V\right)=\lambda_{l}\left(V\right)-1$.

In bending-type DEFAs, the silicone tube is pre-stretched and attached to a flat, flexible, but not extensible substrate, in this study polyimide (PI) film. The actuator has an equilibrium state at V=0 due to the pre-stretch, where the strain energy of the tube and the bending energy of substrate are balanced. An application of voltage (V>0) generates the electrostatic energy and reduced the strain energy, resulting in a bending deformation towards the flat shape. The length of the tube, l, in a bending state is expressed in Equation (11) using the radius of curvature ρ (see Figure 1d).

(11) $$l=\left(\rho +r_{2}\right)\theta =l_{\mathrm{PI}}-r_{2}\theta ,$$

where $l_{\mathrm{PI}}$ and θ denote the length of PI substrate and the bending angle, respectively. Equation (11) becomes Equation (12), considering the incompressibility (Equation (4)), pre-stretch ratio $\lambda_{p}$, and the relationship, $\lambda_{p}=l_{\mathrm{PI}}/l_{0}$.

(12) $$l+r_{20}\theta \sqrt{\frac{l_{\mathrm{PI}}}{\lambda_{p}l}}-l_{\mathrm{PI}}=0.$$

The solution of Equation (12) gives l as a function of θ, i.e., $\lambda_{l}(=l/l_{0})$ is also the function of θ. We assumed that l represents the averaged elongation of the silicone tube. The aqueous electrode was filled after pre-stretching the tube during the fabrication process, which is discussed in Section 3. Therefore, we employed two equations for the strain energy, one for the tube, and one for the aqueous electrode. The latter was considered as a cylinder made of the silicone elastomer having the inner radius of the tube, $r_{1}$. The strain energy of the tube, $U_{\mathrm{strain}\_t}$, and that of the cylinder, $U_{\mathrm{strain}\_c}$, are defined as follows:

(13) $$U_{\mathrm{strain}\_t}=2\pi r_{0}d_{0}l_{0}\overset{3}{\underset{i=1}{\sum }}C_{i}{\left(\lambda_{l}^{2}+\frac{2}{\lambda_{l}}-3\right)}^{i},$$

(14) $$U_{\mathrm{strain}\_c}=\pi r_{10}^{2}l_{0}\overset{3}{\underset{i=1}{\sum }}C_{i}{\left(\frac{\lambda_{l}^{2}}{\lambda_{p}^{2}}+\frac{2\lambda_{p}}{\lambda_{l}}-3\right)}^{i}.$$

Bending energy in the actuator is stored in the substrate. Additionally, the energy is also stored in the adhesive layer used to attach the silicone tube to the substrate. The bending energy of the substrate, $U_{\mathrm{PI}}$, is expressed as follows:

(15) $$U_{\mathrm{PI}}=\frac{1}{2}K_{\mathrm{PI}}\theta^{2}=\frac{1}{2}Y_{\mathrm{PI}}I_{\mathrm{PI}}\theta^{2}=\frac{Y_{\mathrm{PI}}w_{\mathrm{PI}}h_{\mathrm{PI}}^{3}}{24l_{\mathrm{PI}}}\theta^{2},$$

where $K_{\mathrm{PI}}$ denotes the bending stiffness, $Y_{\mathrm{PI}}$ represents Young’s modulus, $I_{\mathrm{PI}}$ is the second moment of inertia, $w_{\mathrm{PI}}$ denotes width, and $h_{\mathrm{PI}}$ is the thickness of the substrate. Similarly, the bending energy of the adhesive layer is as follows:

(16) $$U_{\mathrm{AD}}=\frac{1}{2}K_{\mathrm{AD}}\theta^{2}=\frac{1}{2}Y_{\mathrm{AD}}I_{\mathrm{AD}}\theta^{2}=\frac{Y_{\mathrm{AD}}w_{\mathrm{AD}}h_{\mathrm{AD}}^{3}}{24l_{\mathrm{PI}}}\theta^{2},$$

where $K_{\mathrm{AD}}$ denotes the bending stiffness, $Y_{\mathrm{AD}}$ represents Young’s modulus, $I_{\mathrm{AD}}$ denotes the second moment of inertia, $w_{\mathrm{AD}}$ is the width, and $h_{\mathrm{AD}}$ is the thickness of the adhesive layer. As described in Section 3, we used a sealant (Dowsil 734, Dow Corning) as the adhesive layer.

The electrostatic energy $U_{\mathrm{electric}}$ takes a form same as Equation (7). The total energy in the actuator is given as the sum of energies expressed by Equations (7) and (13)–(16):

(17) $$U_{\mathrm{total}}=U_{\mathrm{strain}\_t}+U_{\mathrm{strain}\_c}+U_{\mathrm{PI}}+U_{\mathrm{AD}}+U_{\mathrm{electric}}.$$

The following equation is considered under the equilibrium state:

(18) $$\frac{\partial U_{\mathrm{total}}}{\partial \theta }=0.$$

The solution of Equation (18) gives θ as a function of V. Table 1 summarizes the model parameters used to design and calculate the theoretical value of DEFAs fabricated in this study.

## 3. Materials and Methods

### 3.1. Fabrication of Silicone Tube

**Figure 2:**
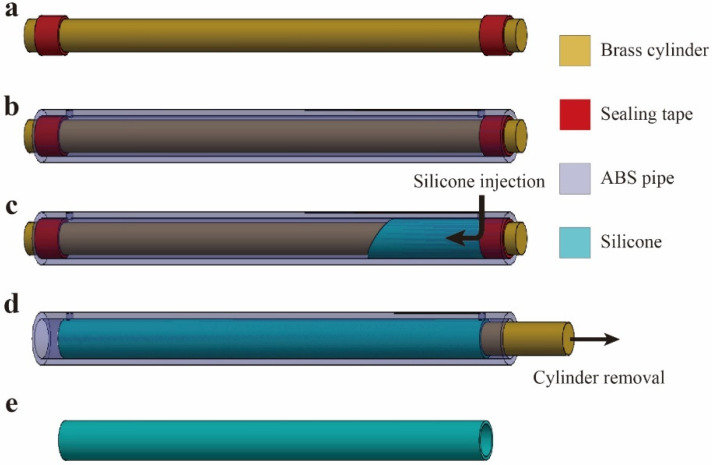
Fabrication process of silicone tube for DEFAs. (**a**) Inner mold made of a brass cylinder and sealing tape. (**b**) Outer mold made of ABS that contains the inner mold. (**c**) Injection of silicone mixture to the mold using a syringe, followed by curing in an oven. (**d**) Removal of the inner mold. (**e**) Silicone tube after taking off from the outer mold.

The fabrication process of the silicone tube followed the method presented in the reference [35]. A mixture of Ecoflex 00–30 (Smooth-On, Macungie, PA, USA) and Sylgard 184 (Dow Corning, Midland, MI, USA) was used as the material for the silicone tube. These silicones were mixed in the liquid state with a weight ratio Ecoflex 00–30:Sylgard 184 of 11:9 using a planetary centrifugal mixer (ARE-310, THINKY, Tokyo, Japan). The silicone mix was determined based on the ratios recommended by the manufacturer: The ratio of the base polymers A:B for Ecoflex 00–30 was 1:1. The ratio of the crosslinking agent to base polymer for Sylgard 184 was 1:10. The mixture was placed in a syringe and injected into a mold made of a brass cylinder (outer diameter of 5 mm), a sealing tape, and an acrylonitrile butadiene styrene (ABS) pipe (inner diameter of 6 mm) (see Figure 2a–c). The length of the entire mold was 220 mm. A release agent was sprayed on the brass cylinder prior to assembling the mold to ensure the removal of the cured silicone tube in the later stage of the fabrication process.

After that, the silicone mixture was cured in an oven at 40 °C for 8 h. The sealing tape was then removed, and a small amount of ethanol was injected into the gap between the outer and inner molds to remove the inner mold (brass cylinder) without causing any damage to the silicone. After removing the inner mold (Figure 2d), the silicone tube was carefully taken off from the inside of the outer mold; it was washed with ethanol to clean the remaining release agent from the surface (Figure 1e). Every fabricated tube was air-pressured using a syringe to confirm that there were no defects, such as uneven thickness or holes. The inner and outer diameters and concentricity of the fabricated silicone tubes were checked using a microscope. There were three samples. The inner diameter, outer diameter, and concentricity were 4.94 ± 0.02 mm, 6.05 ± 0.02 mm, and 0.06 ± 0.03 mm, respectively. In the tensile test, as described in Supplementary Materials and shown in Figure S1, the Young’s modulus of the silicone mixture after curing was 1.26 MPa. This value is reasonable as Ecoflex 00–30 and Sylgard 184 have moduli of 0.1 MPa and 1.75 MPa (at a curing temperature 40 °C), respectively [33,36]. The elongation at break of the silicone mixture after curing was ~220%, intermediate to those of Ecoflex 00–30 (835% [33]) and Sylgard 184 (85% [36]).

### 3.2. Fabrication of DEFAs

**Figure 3:**
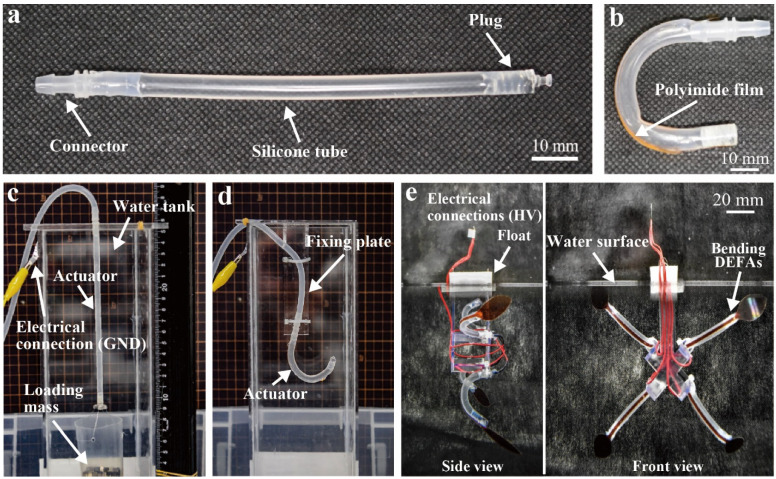
(**a**) Fabricated linear-type DEFA (without aqueous electrode). (**b**) Fabricated bending-type DEFA (without aqueous electrode). (**c**) Experimental setup to characterize linear-type DEFA and (**d**) bending-type DEFA. (**e**) Jellyfish-type underwater robot using four bending-type DEFAs.

The fabrication process of linear-type DEFAs is as follows. The fabricated silicone tube was cut at a length of 100 mm. A sealing plug was then attached to one end, and a connector was attached to the other end. Both connectors were glued to the silicone tube using a sealant (Dowsil 734, Dow Corning). The sealing plug was designed by CAD software (Fusion 360, Autodesk, San Rafael, CA, USA) and fabricated using a 3D printer (Form 3, Formlabs, Somerville, MA, USA). The entire sample was placed in an oven at 40 °C for 10 min to cure the glue. Figure 3a shows the fabricated linear-type DEFA. The effective length of the actuator (i.e., the length of the tube sandwiched between the electrode and the surrounding water) was 75.0 mm. We note that the aqueous electrode was not filled at this stage. The linear-type DEFA was fixed onto a jig with a pre-stretch ratio of 1.3 as the bending-type DEFA. Furthermore, a PI film with a thickness of 50 µm and width of 1.0 mm was adhered to one side of the tube by using a sealant (Dowsil 734, Dow Corning) and cured at 40 °C for 10 min. The sample was then removed from the jig. The fabricated bending-type DEFAs are shown in Figure 3b. Note that the aqueous electrode was not filled at this stage. Table 1 summarizes the dimensions of the fabricated DEFAs.

### 3.3. Characterization of DEFAs

**Figure 4:**
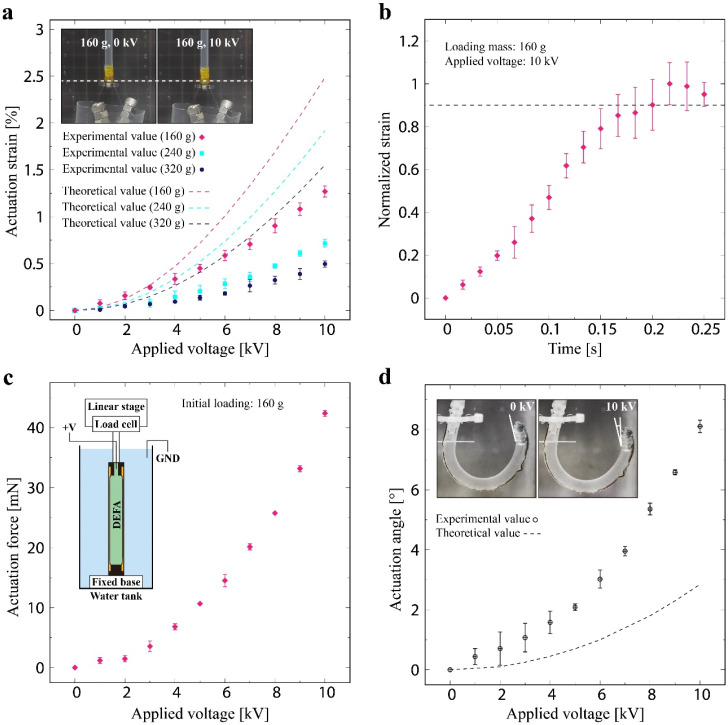
(**a**) Actuation strain of the linear-type DEFA as a function of the applied voltage. (**b**) Response time of the linear-type DEFA (loading mass of 160 g, applied voltage 10 kV). (**c**) Actuation force as a function of the applied voltage. (**d**) Actuation angle of the bending-type DEFA as a function of the applied voltage.

The linear-type DEFA was suspended in a water tank filled with tap water. Loading mass was attached to the actuator tip, as shown in Figure 3c. Loading masses, 160 g, 240 g, and 320 g, were used during the characterization. The other end of the actuator was connected to a silicone tube from which a sodium chloride solution of 16% concentration was filled using a syringe. This additional tube was sealed with a syringe head and an electrical wire for applying high voltage. The input voltage was generated using a function generator (ek-FGJ, Matsusada Precision, Tokyo, Japan) and a DC/DC converter (CB101, XP Power, Singapore), generating the voltages ranging from 0 to 10 kV. The ground side of the converter was immersed in the water tank using conductive tape. Furthermore, a ruler was placed next to the water tank to measure the elongation using a camera (D7500, Nikon, Tokyo, Japan). The characterization of actuation strain as a function of the applied voltage using the above-mentioned setup was performed in the range of 0 to 10 kV with 1 kV steps of increment. The measurement was performed three times for each applied voltage, and the average was calculated. In the same setup, the response time of the actuator at 10 kV and a loading mass of 160 g was measured using the camera. The response time was the time at which 90% of the maximum strain was reached. Furthermore, the actuation force of the linear-type DEFA was characterized by fixing one end of the actuator at the bottom of the water tank, with the other side attached to a load cell (FSH03873, FUTEK, Irvine, CA, USA), as shown in the inset of Figure 4c. The load cell was connected to a linear stage to ensure that the desired initial loading was applied. The initial loading was set as 160 g and a voltage of up to 10 kV was applied in increments of 1 kV. Measurements were obtained three times for each applied voltage, and the average value was calculated.

To characterize the bending-type DEFA, it was fixed in the water tank by using an acrylic plate (Figure 3d). Moreover, the other end of the actuator was connected to a silicone tube from which a sodium chloride solution of 16% concentration was filled using a syringe for establishing an electrical connection to apply high-voltage. The actuation angle was obtained as the change of the tip angle from the initial state. The measurement was performed three times for each applied voltage, and the average was calculated.

### 3.4. Fabrication and Testing of Jellyfish-Type Underwater Robot

The jellyfish-type robot (Figure 3e) consisted of four bending-type DEFAs made of a 60 mm-long silicone tube. These actuators used 125 µm-thick and 3 mm-wide PI film to adjust the initial bending angle, while the pre-stretch ratio was kept at 1.3. The PI film had a fin shape in the part that did not touch the silicone tube for producing thrust forces. The actuators were attached to a frame fabricated through the 3D printer, and a float was attached for the balancing purpose. The connector was sealed with an electrical wire, the sealant, and a shrink tube in every actuator. The electrical connection was established by attaching a thin enamel wire with a diameter of 10 µm. Such a thin wire minimizes its influence of mechanical resistance on the movement of a robot. The setup to apply the voltage and measure the motion was the same as the one used for the characterization of DEFAs, except the water tank, which was larger such that the robot was able to locomote longer distances. We measured the swimming speed of the robot at 10 kV with a driving frequency of 4 Hz. We note that all the actuators in the robot were activated simultaneously.

## 4. Results and Discussion

### 4.1. DEFAs

We observed voltage-controllable elongation of the linear-type DEFA. The actuation strain increased with increasing voltage (see Figure 4a). This trend was the same for all the loading conditions. The experimental data showed a quadratic increase that can be expressed based on the Maxwell stress, $\varepsilon_{0}\varepsilon_{r}{\left(V/d\right)}^{2}$—a fundamental principle commonly used in DEAs. The maximum actuation strain in the tested voltage range was 1.3 ± 0.1% at 10 kV for the loading mass of 160 g. The result also shows that the loading mass influences the actuation strain. The heavier the mass, the smaller the actuation strain because the silicone tube was stiffened in the loading direction reducing the resulting actuation strain. The theoretical values calculated from Equation (10) displays similar trend, meaning that it predicts the behavior of the actuator under stiffening effect. However, the theoretical values were larger than the experimental data. The discrepancy may have resulted from injecting the aqueous electrode manually, leading to a difference in the inner volume of the actuator. An increased inner volume may result in a smaller actuation strain because the contribution of the strain energy expressed in Equation (5) becomes larger, i.e., it causes additional stiffening of the actuator. As shown in Figure 4b, the response time of the linear-type DEFA was 0.2 s. The maximum actuation force in the tested voltage range was 42.4 ± 0.5 mN, as displayed in Figure 4c. The force exhibited quadratic growth resulted from the Maxwell stress, as in the case of the actuation strain.

Voltage-controllable angular displacement was observed for the bending-type DEFA, as shown in Figure 4d. The experimental data showed quadratic growth, such as linear-type DEFA. The maximum actuation angle in the tested voltage range was 8.1 ± 0.2° at 10 kV. The theoretical value calculated from Equation (18) and the parameters in Table 1 well predict the trend; however, there is a discrepancy in the experimental data. The reason may be the same as that of the linear-type DEFA. Here, the internal volume is again different (in this case smaller) from what is expected in the model. A smaller volume may result in a larger actuation angle than the prediction because the contribution of the strain energy (see Equation (14)) becomes smaller than the designed value. One potential solution to reduce the discrepancies between the experimental and theoretical values, observed in both linear and bending-type DEFA, is to use a dispenser machine while injecting the electrode material to precisely control the inner volume.

The value of actuation strain observed from the linear-type DEFA (1.3% at ~20 V/µm) is comparable or even better than other DEAs of similar types reported in the literature (~0.5% at ~40 V/µm [23], ~1.8% at ~12 V/µm [24], and ~1% at ~20 V/µm [26]). This may result from using the surrounding water as the ground electrode, avoiding a dedicated electrode layer that normally acts as a passive component. We note that the magnitude of actuation in DEFA can be amplified by exploiting the resonance of the structure, as shown in Supplementary Video S1. In the video, the actuation strain was ~6.0% (10 kV and 2 Hz), and the actuation angle was ~16° (10 kV and 4 Hz).

A larger actuation strain is expected for DEFAs when applying higher voltage below the breakdown strength of the silicone elastomers used in the actuator (59 V/µm for Ecoflex 00–30 and 100 V/µm for Sylgard 184, as per the reference [33]). Thinning the wall of the silicone tube provides the same effect when the applied voltage is limited. The minimum achievable thickness in this study is limited by the available diameter of the materials used for the mold, the brass cylinder, and the ABS pipe.

The results demonstrate the successful implementation of the actuator notion proposed in this study and suggest the usefulness of the model.

### 4.2. Jellyfish-Type Underwater Robot

**Figure 5:**
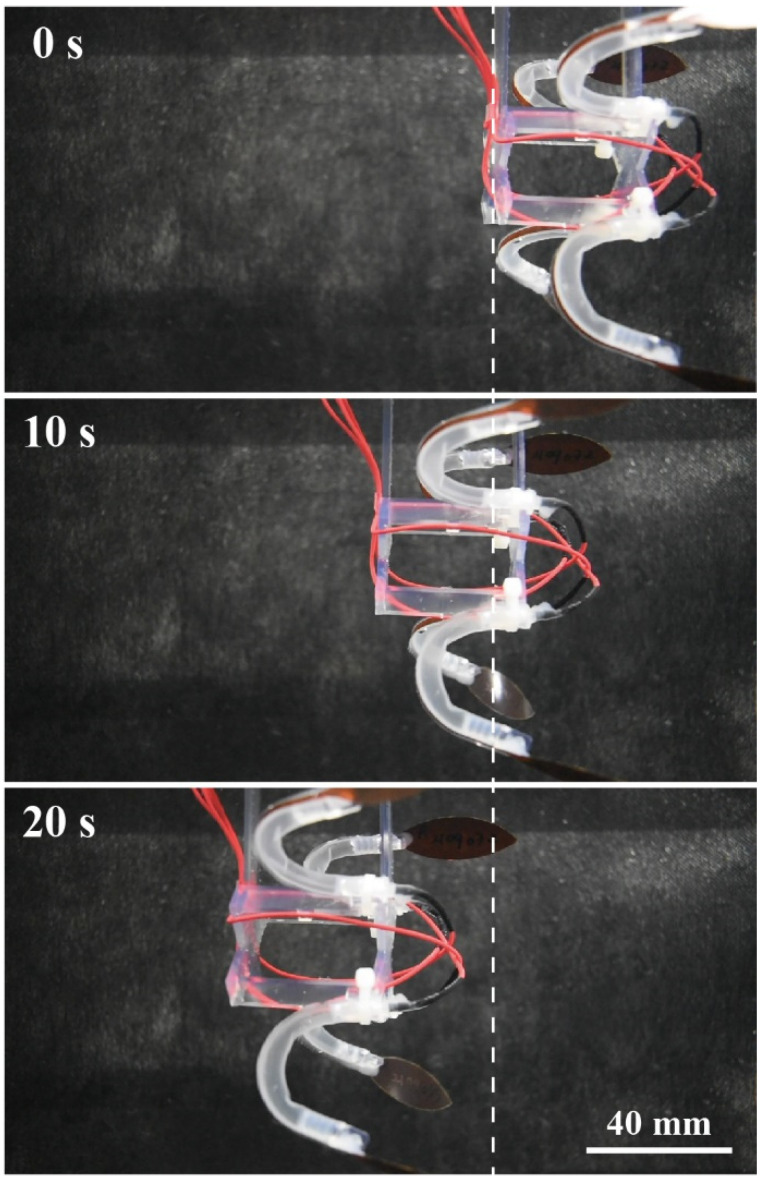
Swimming sequence of the jellyfish-type underwater robot driven at 10 kV and 4 Hz.

The swimming motion of the robot is shown in Figure 5 in chronological order. Furthermore, Video S1 displays the movement of the robot. The swimming speed in the tested condition (10 kV and 4 Hz) was observed to be 3.1 mm/s. This value is comparable to other jellyfish-type robots reported in the literature (~1.5 mm/s at 3 kV and 2 Hz [37] and 1.8 mm/s at 7 kV 0.2 Hz [38]; both were in tethered condition). The swimming performance varies with the applied voltage and driving frequency. We chose the driving frequency of 4 Hz because it resonates with the DEFAs in the robot, maximizing the speed for the tested voltage. In addition, the body-length (BL)/s is used for biomimetic underwater robots to quantify their swimming speed in a normalized fashion [39]. To improve or optimize the design of our robot, characterization should also be performed based on this metric to clarify how far the proposed actuators are effective in underwater systems. Nevertheless, we believe that our result already satisfies the objective of this study because our focus is to demonstrate the applicability of the proposed actuator for soft robotics.

## 5. Conclusions

We developed dielectric elastomer fiber actuators (DEFAs) that work with an aqueous electrode and water environment. We fabricated and characterized linear-type DEFA and bending-type DEFA. The proposed actuator is successfully implemented, as indicated by the results of the voltage-controllable actuation strain, force, and angle. The observed actuation strain is comparable to or even better than other DEAs of similar type. Our analytical model well predicts the behavior of the actuators. However, it also shows a discrepancy in the experimental data, which may result from the inner volume difference of the actuators caused by a manual injection of the aqueous electrode. One potential solution to this problem is to use a dispensing machine that allows to precisely inject the electrode of the designed amount. We built a jellyfish-type robot to demonstrate the applicability of DEFAs for soft robotics. The robot showed swimming speed comparable to other DEA-based robots of the same type.

Future work will focus on further clarifying the actuation characteristics of DEFAs while exploring material combinations for the silicone tube and the substrate to optimize the performance. The understanding of the limitation of the fabrication process based on achievable device size (e.g., the wall thickness) will add insight on how far the actuation performance can be improved and how the device can be scaled. Dynamic response, especially resonance actuation, should also be investigated along with the modification of the analytical model, which will allow designing the actuators and soft robotic systems. Since the shape of DEFA, fiber, could be tailored in various forms, the development of soft robotic systems specialized for using underwater is expected to shed light on the range of practical applications.

## Figures and Tables

**Table 1:** Model parameter of DEFAs.

Parameter	Value	Parameter	Value
Dimensions		Material properties	
Silicone elastomer tube		Silicone elastomer tube	
$\mathrm{Initial}\;\mathrm{wall}\;\mathrm{thickness}\;d_{0}$	0.5 mm	$\mathrm{Dielectric}\;\mathrm{constant}\;\varepsilon_{r}$	3.265 ^2^
$\mathrm{Initial}\;\mathrm{length}\;l_{0}$	75.0 mm	$\mathrm{Material}\;\mathrm{Constant}\;C_{1}$	0.21 MPa ^3^
$\mathrm{Initial}\;\mathrm{radius}\;r_{0}$	2.75 mm	$\mathrm{Material}\;\mathrm{Constant}\;C_{2}$	0.08 MPa
$\mathrm{Initial}\;\mathrm{outer}\;\mathrm{radius}\;r_{20}$	3.0 mm	$\mathrm{Material}\;\mathrm{Constant}\;C_{3}$	2.8 × 10^−8^ MPa
$\mathrm{Initial}\;\mathrm{inner}\;\mathrm{radius}\;r_{10}$	2.5 mm	Polyimide substrate	
Polyimide substrate		$\mathrm{Young}’s\;\mathrm{modulus}\;Y_{\mathrm{PI}}$	3.4 GPa
$\mathrm{Length}\;l_{\mathrm{PI}}$	97.5 mm	Adhesive layer	
$\mathrm{Width}\;w_{\mathrm{PI}}$	1.0 mm	$\mathrm{Young}’s\;\mathrm{modulus}\;Y_{\mathrm{AD}}$	0.48 MPa ^4^
$\mathrm{Thickness}\;h_{\mathrm{PI}}$	50 µm	Other parameters	
Adhesive layer ^1^		$\mathrm{Pre}-\mathrm{stretch}\;\mathrm{ratio}\;\lambda_{p}$	1.3
$\mathrm{Thickness}\;h_{\mathrm{AD}}$	0.25 mm	$\mathrm{Permittivity}\;\mathrm{of}\;\mathrm{free}\;\mathrm{space}\;\varepsilon_{0}$	8.85 × 10^−12^ F/m

^1^ Length and width of the adhesive layer are the same as those of the polyimide substrate. ^2^ Estimated value based on the ratio of Ecoflex 00–30 and Sylgard 184 and their dielectric constant at 1 Hz, which is available in [33]. ^3^ Material constants were obtained through the tensile test, as described in Supplementary Materials. ^4^ Estimated value based on the datasheet of DOWSIL 734, which is available in [34].

## Data Availability

Not applicable.

## Supplementary Materials

The following are available online at https://www.mdpi.com/article/10.3390/polym13244310/s1, Video S1: Resonant actuation of DEFAs and swimming of jellyfish-type robot. Figure S1. Stress-strain behavior of the silicone elastomer material used in this study. Note that the strain is represented as stretch.
