# Supplementary material for: Dielectric Elastomer Fiber Actuators with Aqueous Electrode
Source: Polymers (Basel). 2021 Dec 9;13(24):4310. doi: 10.3390/polym13244310 (PMC8708416; doi:10.3390/polym13244310)
Supplement: Supplementary file 1 [file polymers-13-04310-s001.zip › polymers-1465740 - Supplementary material-done.pdf]

polymers

Supplementary Material

Dielectric Elastomer Fiber Actuators with Aqueous Electrode

Keita Shimizu, Toshiaki Nagai and Jun Shintake *

Department of Mechanical and Intelligent Systems Engineering, Graduate School of Informatics and Engineering, University of Electro-Communications, 1-5-1 Chofu-gaoka, Chofu, Tokyo 182-8585, Japan; s2132048@edu.cc.uec.ac.jp (K.S.); n2032077@edu.cc.uec.ac.jp (T.N.)
* Correspondence: shintake@uec.ac.jp; Tel.: +81-42-443-5393

Tensile test of the silicone elastomer

Ecoflex 00-30 (Smooth-On) and Sylgard 184 (Dow Corning) were mixed in the liquid state with a weight ratio of Ecoflex 00-30:Sylgard 184 = 11:9 using a planetary centrifugal mixer (ARE-310, THINKY). The mixture was then blade-casted on a Polyethylene terephthalate (PET) and cured in an oven at 40°C for 8 h, forming a membrane. The membrane was then punched out into five samples with a dumbbell shape (JIS K6251, ISO 37, Type 1A). The average thickness of the samples was 1.07 ± 0.10 mm, which was measured by a laser displacement sensor (CDX-L15, OPTEX FA). Then, uniaxial tensile was performed on all the samples using a universal testing machine (AGS-20NX, Shimadzu) from which the stress–strain curves were recorded. This test was performed at a tensile speed of 50 mm/min until the sample broke. By fitting Equation (6) in the main manuscript to the measured stress–strain data, the material constants of Yeoh hyperelastic material model C_1, C_2, and C_3 are obtained. Figure S1 plots the measured stress-strain data and the fitted curve. Additionally, Young's modulus Y was calculated using the relationship $Y = 6C_1$.

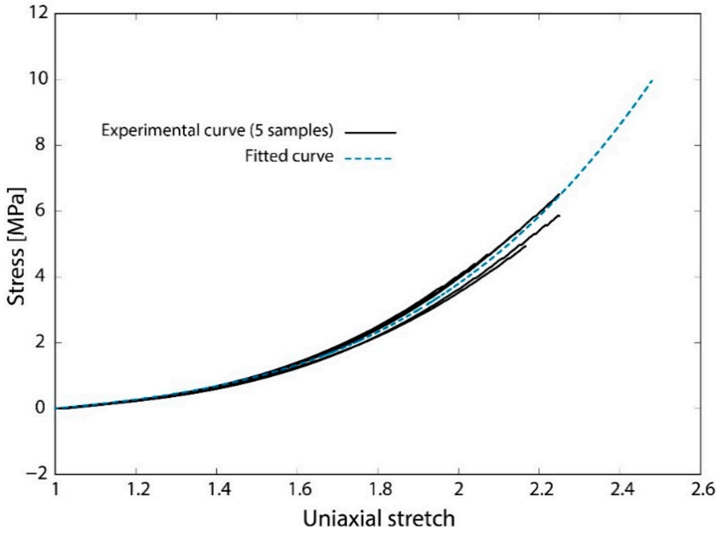

Figure S1. Stress-strain behavior of the silicone elastomer material used in this study. Note that the strain is represented as stretch.
